# Brain structure in autoimmune Addison’s disease

**DOI:** 10.1093/cercor/bhac389

**Published:** 2022-10-13

**Authors:** Annelies van’t Westeinde, Nelly Padilla, Monica Siqueiros Sanchez, Sara Fletcher-Sandersjöö, Olle Kämpe, Sophie Bensing, Svetlana Lajic

**Affiliations:** Pediatric Endocrinology Unit, Department of Women’s and Children’s Health, Karolinska Institutet, Karolinska University Hospital, Karolinskavagen 37A, SE-171 76 Stockholm, Sweden; Unit for Neonatology, Department of Women’s and Children’s Health, Karolinska Institutet, Karolinska University Hospital, Karolinskavagen 37A, SE-171 76 Stockholm, Sweden; Brain Imaging, Development and Genetics (BRIDGE) Lab, Division of Interdisciplinary Brain Sciences, Department of Psychiatry and Behavioral Sciences, Stanford University School of Medicine, 401 Quarry Road, Stanford, CA 94305-5101, United States; Department of Molecular Medicine and Surgery, Karolinska Institutet, SE-171 76 Stockholm, Sweden; Department of Endocrinology, Karolinska University Hospital, SE-171 76 Stockholm, Sweden; Department of Endocrinology, Karolinska University Hospital, SE-171 76 Stockholm, Sweden; Department of Medicine (Solna), Center for Molecular Medicine, Karolinska Institutet, Karolinska University Hospital, SE-171 76 Stockholm, Sweden; Department of Molecular Medicine and Surgery, Karolinska Institutet, SE-171 76 Stockholm, Sweden; Department of Endocrinology, Karolinska University Hospital, SE-171 76 Stockholm, Sweden; Pediatric Endocrinology Unit, Department of Women’s and Children’s Health, Karolinska Institutet, Karolinska University Hospital, Karolinskavagen 37A, SE-171 76 Stockholm, Sweden

**Keywords:** Addison, brain structure, executive function, working memory, cortisol

## Abstract

Long-term disturbances in cortisol levels might affect brain structure in individuals with autoimmune Addison’s disease (AAD). This study investigated gray and white matter brain structure in a cohort of young adults with AAD. T1- and diffusion-weighted images were acquired for 52 individuals with AAD and 70 healthy controls, aged 19–43 years, using magnetic resonance imaging. Groups were compared on cortical thickness, surface area, cortical gray matter volume, subcortical volume (FreeSurfer), and white matter microstructure (FSL tract-based spatial statistics). Individuals with AAD had 4.3% smaller total brain volume. Correcting for head size, we did not find any regional structural differences, apart from reduced volume of the right superior parietal cortex in males with AAD. Within the patient group, a higher glucocorticoid (GC) replacement dose was associated with smaller total brain volume and smaller volume of the left lingual gyrus, left rostral anterior cingulate cortex, and right supramarginal gyrus. With the exception of smaller total brain volume and potential sensitivity of the parietal cortex to GC disturbances in men, brain structure seems relatively unaffected in young adults with AAD. However, the association between GC replacement dose and reduced brain volume may be reason for concern and requires follow-up study.

## Introduction

Due to destruction of the adrenal cortex, individuals with autoimmune Addison’s disease (AAD) have chronic glucocorticoid (GC) and mineralocorticoid (MC) deficiency and lack adrenal androgens (Charmandari et al. 2014). In females, this lack of adrenal androgens results in at least 50% reduction of total androgen levels (Allolio et al. 2007). AAD usually has its onset in young adulthood or middle age and has a slightly higher prevalence in women, at least for those older than 30 years (Erichsen et al. 2009; Bensing et al. 2016; Martin-Grace et al. 2020). Comorbidity with other autoimmune diseases is common, in particular thyroid disorders (Betterle and Morlin 2011; Pazderska and Pearce 2017; Martin-Grace et al. 2020). Individuals with AAD are treated life-long with replacement medication for GC's and MC's. This treatment usually consists of oral immediate release hydrocortisone (IR-HC) administered 2-3 times per day and fludrocortisone administered once per day (Løvås and Husebye 2003). Androgens are not replaced as part of the standard treatment but may sometimes be given to women (Bornstein et al. 2016).

In healthy individuals, GCs follow a diurnal and ultradian rhythmic secretion pattern that is hard to mimic with oral replacement (Choudhury et al. 2019). Individuals with AAD lack the morning rise in cortisol, have much fewer pulses throughout the day, and may experience too high cortisol levels in the afternoon (Choudhury et al. 2019). In addition, the hypothalamus–pituitary–adrenal axis is rendered less flexible, as it cannot produce cortisol in response to internal and external stressors. Thus, individuals with AAD experience periods of supra- and infra-physiological cortisol levels, which may disrupt numerous physiological functions, including the sleep cycle (Vargas et al. 2018; Choudhury et al. 2019). Oral modified-release hydrocortisone (MR-HC) formulas aim to reduce the extremities in cortisol peaks and troughs, but nonetheless further diminish ultradian rhythmicity (Whitaker et al. 2014; Stewart 2019).

Both short- and long-term disruptions in cortisol levels and rhythmicity might affect brain structure development (McEwen et al. 1986; Shirazi et al. 2015; Joëls 2018). Animal studies have shown that both pre- and postnatal disturbances in cortisol affect neuronal growth, dendritic arborization, and long-term potentiation, although the effects depend on GC dose, brain region, and developmental time-window (Shirazi et al. 2015). For example, GCs might impair neurogenesis when given in high doses while stimulating neurogenesis at intermediate doses (Shirazi et al. 2015; Stomby et al. 2016; Morsi et al. 2018). The hippocampus and the prefrontal cortex are particularly sensitive to fluctuations in GC levels due to their high glucocorticoid receptor (GR) and mineralocorticoid receptor (MR) density (Reul and de Kloet 1985; Lupien et al. 1999; McEwen and Magarinos 2001; Joëls 2008; Madalena and Lerch 2017). For example, individuals with Cushing’s disease, who are exposed to excessive cortisol production for a prolonged period, have reduced hippocampal volumes among other (Andela et al. 2015). Moreover, individuals with congenital adrenal hyperplasia (CAH), who also experience life-long GC and MC deficiency and who are treated with the same medication as individuals with AAD, have altered structure of the brain as adults, particularly in the frontoparietal network and cerebellum that are partly related to reduced working memory performance (Karlsson et al. 2017; Herting et al. 2020; Van't Westeinde et al. 2020). Thus, the effects of long-term cortisol disturbances on brain structure may be widely distributed and not only depend on the expression pattern of GRs and MRs in the brain but may for example also be influenced by regional differences in energetic demand (Liu et al. 2018). In addition to gray matter, cortisol disturbances may also affect white matter. Oligodendrocytes express GRs and need cortisol to develop (Barres et al. 1994; Matsusue et al. 2014). Both Cushing’s disease and CAH have been associated with changes in white matter microstructure, with individuals with CAH showing more white matter abnormalities compared to controls (Mnif et al. 2013; Andela et al. 2015; Webb et al. 2018; Van’t Westeinde et al. 2020; Cotter et al. 2021). In addition to direct effects of cortisol via GRs, alterations in inflammatory processes may also impact white matter microstructure and mediate these effects (Altendahl et al. 2020). Disturbances in cortisol in individuals with AAD may therefore affect both gray and white matter. However, it is important to consider the age at diagnosis of patients, as cortisol affects the brain differentially depending on the developmental time window of exposure (Lupien et al. 2009). Different effects in terms of brain structure might therefore be found depending when the patients were diagnosed.

In addition to affecting structural development, cortisol is involved in memory formation, selective attention, and learning and further helps to control sleep, motivation, mood, and fear (Lupien and McEwen 1997; Belanoff et al. 2001; Lupien et al. 2005; Hannibal and Bishop 2014; Kalafatakis et al. 2018). These complex processes require precisely regulated GC levels, in which the dynamic hormonal oscillations under natural conditions are crucial (Herbert et al. 2006; Oster et al. 2017; Kalafatakis et al. 2018; Kalafatakis et al. 2019; Kalafatakis et al. 2021) (De Kloet et al. 1998; Gallagher et al. 2009). In addition to GCs, the brain is sensitive to androgen levels. Dehydroepiandrosterone (DHEA) seems to affect structural brain development in a sex-dependent manner and is associated with working memory performance and emotion regulation (Nguyen et al. 2013; Nguyen et al. 2017; Farooqi et al. 2018). Interestingly, the ratio between cortisol and DHEA seems to be especially relevant for optimal brain function and development (Farooqi et al. 2018).

Thus far, no brain imaging studies have been conducted on individuals with AAD. While patients with CAH are known to experience problems with cognitive function, particularly in working memory (Karlsson et al. 2017), this seems to be less pronounced in individuals with AAD. Reduced verbal memory and impairments in executive functions are, however, sometimes reported in AAD (Henry et al. 2014; Schultebraucks et al. 2015; Tiemensma et al. 2016; Henry et al. 2017) and may be related to sleep disruptions (Henry et al. 2017), illness duration, and number of adrenal crises (Henry et al. 2014). In a previous study, we found that, overall, young adults with AAD performed well on cognitive tests, although females reported more executive functioning-related problems in daily life, which in turn were associated with mental fatigue and a lower medication dose ([Bibr ref1][Bibr ref1]). Nonetheless, the long-term GC disturbance could still influence structural brain development, which in turn might contribute to the experience of executive function problems, despite the relatively good performance on cognitive tests. Thus, in the present study, we sought to investigate brain structure in a cohort of young adults with AAD, using a whole-brain exploratory approach of gray matter and white matter microstructure. We chose an exploratory approach as this is a first study of brain structure in this patient population. In addition, we aimed to investigate the modulating effect of sex. Finally, we tested the association between brain structure, disease-related factors, self-reported executive function problems, and performance on working memory tests.

## Material and methods

### Participants

Participants with AAD and controls were recruited as part of a larger study on primary adrenal insufficiency (CAH and AAD) in the Swedish population (Karlsson et al. 2017). All participants were Caucasian. Participants were free from alcohol or drug abuse and did not have any magnetic resonance imaging (MRI) contraindications. Individuals with AAD were recruited via the Swedish Addison Registry (Dalin et al. 2017). They were 18–45 years old, diagnosed at least 2 years ago, and had tested positive for 21-hydroxylase autoantibodies (Winqvist et al. 1992; Husebye et al. 2021). Exclusion criteria for individuals with ADD were autoimmune polyglandular syndrome type 1 (APS-1), diabetes type 1, epilepsy, and a history of severe psychiatric problems, as established via a telephone interview. We allowed mild depression, anxiety, or attention deficit hyperactivity disorder, and co-morbid hypothyroidism in the patient group, as these were of interest to study. Control participants were between 18 and 45 years of age; were not treated with GCs; did not have any autoimmune disease or psychiatric problems in the past or present; had normal fasting levels of cholesterol, triglycerides, and insulin; and normal blood pressure. Seven controls and 6 individuals with AAD did not take part in the MRI. After MRI-scan quality control, the final sample consisted of 52 (33 females) individuals with AAD and 70 (39 females) controls, aged 19–43 years. Fourteen individuals had been diagnosed with AAD before 18 years of age (mean age at diagnosis = 15.6, SD = 1.4). Twenty-two individuals with AAD had co-morbid hypothyroidism (Supplementary Table 1). Forty-three patients were treated with IR-HC (2 or 3 daily doses) and 9 with MR-HC (Plenadren) once daily. All patients except one received replacement with 9-α fludrocortisone (Florinef). Seven females received DHEA treatment, of which 2 had premature ovarian insufficiency. Six individuals with AAD and 9 controls were on contraceptive pills. All participants gave written informed consent to take part in the study. The study was approved by the Regional Ethical Committee of Karolinska Institutet and by the Swedish Ethical Review Authority (dnr 99-153, 2011/1764-32, 20140917, 2017/1658-32, 2018/1037-32, 2020-00564).

### Procedures

Participants underwent MRI of the brain, performed neuropsychological tests administered by a trained psychologist, and filled out a series of self-report questionnaires—for more details please refer to van’t Westeinde et al. (2022). In the present study, we assessed working memory using the following measures: the Span Board Test forward and backward (Wechsler 2008) for visuospatial working memory performance (*T*-scores, population norm *M* = 10, SD = 3) and Digit Span from the Wechsler Adult Intelligence Scale (WAIS-IV; Wechsler 2008) for verbal working memory (*T*-scores). We also assessed experienced difficulty with executive function in the past 2 weeks using a self-report measure, the Barkley Deficits in Executive Functioning Scale, short form (BDEFS-SF; Barkley 2011). Self-reported symptoms of depression and anxiety were assessed with the Hospital Anxiety and Depression Scale (HADS) (Pallant and Baily 2005; Zigmond and Snaith, 1983), and symptoms of depression with the Montgomery-Asberg Depression Rating Scale (MADRS) (Montgomery and Asberg, 1979). Participants with AAD also provided disease-related information regarding medication dose and type, time of medication intake, age at diagnosis, and number of adrenal crises since diagnosis (an episode of acute adrenal insufficiency requiring hospital treatment).

#### MRI data acquisition and analyses

MRI scans were acquired on a 3T MR scanner (Discovery MR750, General Electric, Milwaukee, WI, USA) with an 8-channel head coil. For the present paper, we analyzed data from the anatomical T1-weighted image (T1-weighted BRAVO sequence, time repetition [TR] = 7.9 ms, time echo [TE] = 3.1 ms, 176 slices, voxel size: 1.0 × 1.0 × 1.0 mm^3^) and diffusion-weighted scans (TR = 7.4 s, 62 slices, voxel size: 2.3 × 2.3 × 2.3 mm, 60 directions diffusion-weighted images (*b* = 1,500 s/mm^2^), 8 images with no diffusion sensitization (*b* = 0 s/mm^2^)).

#### Analysis of T1 imaging data

We estimated cortical thickness, surface area, gray matter volume, and volumes of subcortical structures with a surface-based approach from T1-weighted images using the FreeSurfer pipeline (v6) (http://surfer.nmr.mgh.harvard.edu/). Cortical reconstruction provided estimates of pial surface (cerebral spinal fluid-gray matter boundary), white matter surface (gray-white matter boundary), and segmentation for subcortical volumetric structures. Errors in the generation of the pial and white matter surfaces were manually fixed by editing the brain masks to remove non-gray matter, the white matter volumes to remove non-white matter, and by adding control points in regions with intensity problems. All participants’ images required manual editing. After the final quality control, 10 participants (1 control and 9 patients) were excluded from the analyses. The surfaced-based data were smoothed using a 10-mm full-width at half-maximum smoothing kernel. Technical details of these procedures have been described previously and are documented on the FreeSurfer website (http://surfer.nmr.mgh.harvard.edu/) (Dale et al. 1999; Fischl et al. 1999). First, we ran a vertex-wise whole-brain analysis using FreeSurfer’s Qdec application. Qdec fits a general linear model at each surface vertex to explain the data, and significant clusters were defined with Monte Carlo simulation using pre-run data in Qdec with 10,000 permutations. Talairach coordinates are reported. Second, we implemented a parcellation-based approach using the Desikan–Killiany atlas. Here the brain is segmented into regions of interest (ROIs), 68 bilateral cortical ROIs and 14 subcortical ROIs. Structural measures are then estimated for each ROI as well as an estimate of total brain volume and intracranial volume (ICV) (Destrieux et al. 2010). False discovery rate (FDR) was applied to correct for multiple comparisons of all atlas-based regions and *q*-values are reported. We opted to include both a vertex-wise and a parcellation approach for 2 reasons. First, a vertex-wise approach provides a more locally precise estimate when assessing structural differences; however, a parcellated approach is more amenable for testing associations between brain and cognitive function/working memory. Second, for robustness, by including two approaches that are differentially sensitive to noise, we can test if findings are consistent across the these two methods of analyzing the data.

#### Analysis of diffusion tensor imaging data

One control participant (female) was excluded from the diffusion-weighted imaging analysis due to bad image quality. Voxel-wise whole-brain tract-based spatial statistics (TBSS) analysis was run to obtain estimates of fractional anisotropy (FA), mean diffusivity (MD), axial diffusivity (AD), and radial diffusivity (RD) (Smith et al. 2006). To correct for eddy currents and motion, we used FSL’s eddy correction method without top-up, but including the -repol option to perform outlier replacement (Andersson et al. 2016). We used rotated bvecs for further steps, brain-extracted Eddy-corrected data using BET (f 0.1), and created FA images by fitting a tensor model to the raw diffusion data using the FMRIB diffusion toolbox (FDT) (Smith 2002). FA data were aligned into a common space (FNIRT, a nonlinear registration tool), using a b-spline representation of the registration warp field (Rueckert et al. 1999). Mean FA images were created and thinned to create a mean FA skeleton representing the centers of all tracts common to the group. Each participant’s aligned FA data were projected onto the skeleton and fed into voxel-wise cross-subject statistics. The nonlinear warps and skeleton projection were also applied to the participants’ MD, AD, and RD images using the tbss_non_FA script. Significant clusters were defined using threshold-free cluster enhancement (TFCE) (Smith and Nichols 2009; Winkler et al. 2014) and permutation testing with 10,000 permutations using FSL’s randomize tool. TFCE-corrected clusters were localized using the Johns Hopkins University White Matter Tractography Atlas.

### Statistical analyses

All statistical analyses were conducted using the open source R software, version 3.6.1 (Team RC 2013). Linear regressions models were used unless otherwise specified, with sex and age as covariates. Results are reported with and without ICV as a covariate in the case of FreeSurfer analyses. Based on a linear regression model with 3 predictors (e.g. group, sex, and age) and *n* = 122 subjects, at alpha 0.05, this study has a power of at least 80% to detect effect sizes of 0.31 or larger (calculated with R’s pwr function).

#### Demographic and disease-specific characteristics of the study group

Group differences were determined between participants with AAD and controls in: proportion of males and females, education level (higher education defined as having completed at least 3 years of university studies), parental education (either of the parents having completed at least 3 years of university studies) (chi-square tests), illegal drug use (Fisher’s exact test), age, and alcohol use (Wilcoxon test for nonparametric data), and among participants with AAD: sex differences regarding hypothyroidism (chi-square tests), total hydrocortisone replacement dose (either IR-HC or MR-HC), total mineralocorticoid replacement dose, number of adrenal crises, age of disease onset, and disease duration (Wilcoxon test for nonparametric data).

#### Group comparisons for brain structure

We compared individuals with AAD to controls on: (i) total brain volume (without ventricles) and ICV; (ii) vertex-wise (FreeSurfer QDEC) and ROI (FreeSurfer Desikan–Killiany) derived cortical thickness, surface area and volume, and subcortical volumes (FreeSurfer Desikan–Killiany); (3) white matter microstructure FA, MD, AD, and RD (FSL-TBSS). Comparisons on these measures were done in 3 steps: (i) whole group comparison, (ii) interaction between AAD and sex, and (iii) post hoc analyses dividing by sex for tests where a significant interaction term was found. Results were considered significant with a *P*- or *q*-value of <0.05 after correction for multiple comparisons.

#### Associations between brain structure, working memory, self-reported executive functions, and disease-related factors

To assess if the relationship between brain structure and cognitive estimates (working memory and executive function) differs between individuals with AAD and controls, we tested the interaction between groups and brain structure estimates (all Desikan–Killiany atlas ROIs, and whole-brain white matter microstructure FA, MD, AD, and RD), with tests of working memory (WMS Span Board tests forward and backward, WAIS digit span), and self-reported problems with executive function (BDEFS-SF total score). For those structural estimates where a significant interaction with group was found, we performed post hoc tests divided by diagnostic group. Linear regression models were used with cognitive estimates as outcome variables, and with sex and age as covariates. The analyses were repeated with and without correcting for ICV.

Within the patient group, we tested if GC replacement dose (HC equivalents, mg/m^2^/day), age at diagnosis, disease duration, and number of adrenal crises (all predictor variables in one model) were associated with brain structure (outcome variables) using linear regression models. Sex and age were corrected for in all models.

#### Subgroup analyses

As autoimmune hypothyroidism was common in the AAD cohort (*n* = 22), we compared individuals with thyroid comorbidity to 22 healthy controls, matched for age and sex, in terms of all brain structure estimates.

## Results

### Demographics

Individuals with AAD were on average 3 years older than controls (*t* = −2.18, *P* = 0.03). There were no group differences in sex distribution, education level, or drug and alcohol use (Table 1). Within the patient group, there were no sex differences in IR-HC or MR-HC use, average GC replacement dose, average MC replacement dose, number of adrenal crises, age at diagnosis, disease duration, or the presence of autoimmune hypothyroidism (Table 2).

**Table 1:** Demographic and background data of all participants.

Variable		AAD (*n* = 52)	Control (*n* = 70)	*P*-value
Sex	% Female	63%	56%	0.500
Age	Mean (SD) Range	32.5 (6.3) 19–42	29.8 (7.5) 19–43	0.031
Education^a^	% Higher Educated	50%	40%	0.360
Parental education^a^	% Higher Educated	42%	57%	0.151
Alcohol^b^	N per week	1.2	1.3	0.447
MADRS	Mean (SD) Range	8.65 (6.11) 0–25	6.87 (5.53) 0–23	0.056
HADS Depression	Mean (SD) Range	3.75 (3.24) 0–11	2.99 (2.65) 0–12	0.112
HADS Anxiety	Mean (SD) Range	7.31 (4.26) 2–20	6.12 (3.53) 0–16	0.057

Wilcoxon test for nonparametric data is applied for group differences in age, well-being, and alcohol use. Chi-square test is applied for group differences in sex and education levels. Linear models were used to test the difference in MADRS, HADS Depression, and HADS Anxiety as these included the covariates sex and age.

^a^Percentage subjects who have completed at least 3 years of University education. For parental education, they were counted as having higher education if either of the parents had completed more than 3 years of University.

^b^Number of times per week the individual used to consume alcohol.

**Table 2:** Medication use and disease characteristics of the individuals with AAD.

Type of GC	All, *n* = 52	Females, *n* = 33	Males, *n* = 19	*P*-value (female vs. male)
IR-HC (*n*)	43	27	16	
GC dose (mg/m^2^/day)^a^ (mean (SD))	13.3 (3.4)	13.3 (3.5)	13.4 (3.4)	0.811
Doses/day (mean (SD))	2.6 (0.7)	2.6 (0.7)	2.6 (0.6)	0.885
MR-HC (*n*)	9	6	3	
GC dose (mg/m^2^/day)^b^ (mean (SD))	12.3 (2.8)	11.2 (2.2)	14.4 (2.9)	0.187
Doses/day (mean (SD))	1.4 (0.7)^c^	1.3 (0.5)	1.7 (1.2)	0.673
9-α fludrocortisone, median dose in mg (range)^d^	0.1 (0.05–0.25)	0.1 (0.05–0.25)	0.1 (0.05–0.20)	0.117
Disease characteristics				
Age at AAD diagnosis (years) (mean (SD)) median range	22.7 (6.3) 22 13–34	23.5 (6.2) 23 13–34	21.7 (6.6) 19 14–34	0.344
AAD duration (years) (mean (SD)) median range	9.6 (5.0) 9.8 2.5–25	9.4 (4.0) 9.8 2.5–18	10.1 (6.6) 9.0 2.8–25	0.668
Number of adrenal crises (mean (SD)) median range	3.4 (5.3) 1 0–25	4 (5.6) 2 0–25	2.5 (4.6) 1 0–20	0.296
Hypothyroidism	42.3%	48.5%	31.6%	0.370
Depression and ADHD				
ADHD diagnosis	1	1	0	1
SSRI use (yes)	7	7	0	0.083

*P*-values indicate comparisons between male and female patients, Wilcoxon tests for nonparametric data were used, and chi-square tests for the relative number of participants with an ADHD diagnosis and using antidepressants. From those on IR-HC replacement therapy, 1 patient had only one dose per day, 19 had 2 doses per day, 20 had 3 doses per day, and 3 individuals had 4 doses per day. ADHD, attention deficit hyperactivity disorder; SSRI, selective serotonin reuptake inhibitor.

^a^Total IR-HC dose per day in mg/m^2^ of body surface (total mg/sqrt (cm^*^kg/3,600)).

^b^Total IR-HC equivalence dose per day in mg/m^2^ of body surface (total mg/sqrt(cm^*^kg/3,600)).

^c^Some participants on MR-HC take additional IR-HC doses on demand.

^d^34 Individuals were on a dose of 0.1 mg of Florinef (9-α fludrocortisone), to replace mineralocorticoids, 5 individuals were on 0.05 mg, 1 patient on 0.075, 1 on 0.2 mg, 1 on 0.25 mg, and 1 patient did not take Florinef.

### Main group comparisons

#### *Total brain volume (covariates*: *age, sex)*

Individuals with AAD had 4.3% smaller total brain volumes (*B* = −35,772, *P* = 0.026) and 4.1% smaller ICVs (*B* = −46134.6, *P* = 0.042). The groups did not differ in height (*B* = −1.79, *P* = 0.072). There were no sex-specific group differences.

#### Cortical thickness, surface area, and cortical and subcortical volumes

##### Covariates: age, sex

Please refer to Table 3 for all coordinates and statistics of the significant findings. In sum, the vertex-wise analyses showed that compared to unaffected controls, participants with AAD had smaller surface area of the left supramarginal gyrus and right inferior parietal cortex and smaller volume of the right lateral orbitofrontal cortex. In addition, there was an interaction between diagnostic group and sex for volume and surface area of the right superior parietal cortex. Post hoc tests revealed that only male participants with AAD had smaller volume and surface area of this region compared to control males. These findings were not replicated using the parcellated-based approach (Desikan–Killiany atlas). However, subthreshold group differences were found, which did not survive FDR correction. These were mostly cortical areas (inferior and superior parietal cortex, supramarginal gyrus, orbitofrontal cortex, and parahippocampal gyri) and the interaction between group and sex for volume of the right supramarginal gyrus (see Table 4, which includes an estimate of % change in size of the regions).

**Table 3:** Results from the vertex-wise analyses comparing participants with AAD to controls in terms of cortical volume, surface area, and thickness.

Region	*P*-value; *t*-max; size in mm^2^ not corrected for ICV	Coordinates cluster peak	*P*-value: *t*-max size in mm^2^ corrected for ICV	Coordinates cluster peak
**All participants with AAD vs. all control participants**				
*Reduced volume in patients* Right lateral orbitofrontal cortex	*P* = 0.01; *t*(max) = 2.04; size = 526.80 mm^2^	*X* = 13.7, *Y* = 32.2, *Z* = −23.2	NS	NS
*Reduced surface area in patients* Left supramarginal gyrus	*P* = 0.05; *t*(max) = 2.55; size = 2231.51 mm^2^	*X* = −49.6, *Y* = −48.0, *Z* = 44.6	NS	NS
*Reduced surface area in patients* Right inferior parietal cortex	*P* = 0.05; *t*(max) = 2.02; size = 1991.62 mm^2^	*X* = 43.2, *Y* = −53.6, *Z* = 42.5	NS	NS
**Interaction between diagnostic group and sex**				
*Volume* Right superior parietal cortex	*P* = 0.01; *t*(max) = 2.72; size = 644.28 mm^2^	*X* = 30.6, *Y* = −47.5, *Z* = 44.4	*P* = 0.01; *t*(max) = 2.10; size = 485.65 mm^2^	*X* = 30.6, *Y* = −47.5, *Z* = 44.4
*Surface area* Right superior parietal cortex	*p* = 0.01; *t*(max) = 2.82; size = 1213.73 mm^2^	*X* = 30.6, *Y* = −47.5, *Z* = 44.4	NS	NS
**Post hoc test comparing participants with AAD to controls, split by sex**				
*Reduced volume in male patients* Right superior parietal cortex	*P* = 0.001; *t*(max) = 2.52; size = 266.23 mm^2^	*X* = 30.7, *Y* = *−*53.3, *Z* = 58.6	*P* = 0.05; *t*(max) = 2.72; size = 1166.51 mm^2^	*X* = 30.6, *Y* = −47.5, *Z* = 44.4
*Reduced surface area in male patients* Right superior parietal cortex	*P* = 0.01; *t*(max) = 4.00; size = 1885.06 mm^2^	*X* = 30.6, *Y* = −47.5, *Z* = 44.4	NS	NS

All models included sex and age at testing as covariates. Findings are presented with and without correction for ICV. NS, not significant.

**Table 4:** Subthreshold group differences (that did not survive multiple comparisons corrections) in cortical volume, surface area and thickness, and subcortical volumes, using the Desikan–Killiany atlas, including sex and age as covariates.

Region	% change in individuals with AAD	*P*-value	*P*-value corrected for ICV
Total brain volume	−4.3	0.026	N/A
ICV	−4.1	0.042	N/A
Cortical volume			
Left fusiform	−6.6	0.017	0.119
Left inferior parietal	−6.6	0.042	0.201
Left isthmus cingulate	−8.1	0.018	0.149
Left pars opercularis	−10.5	**0.009**	**0.040**
Left superior parietal	−6.4	0.044	0.252
Right superior parietal^a^	−6.8	0.039	0.253
Left supramarginal	−10.3	**0.003**	**0.020**
Right supramarginal^a^	−8.4	0.010	0.088
Left parahippocampal	−7.0	0.029	0.099
Right parahippocampal	−5.9	0.035	0.164
Right entorhinal	−7.7	**0.022**	**0.036**
Right medial orbitofrontal	−7.2	**0.003**	**0.031**
Right middle temporal	−6.8	0.034	0.279
Right precentral	−5.9	0.018	0.143
Right precuneus	−6.0	0.045	0.377
Cortical surface area			
Left isthmus cingulate	−7.9	0.012	0.107
Left lateral orbitofrontal	−5.9	0.008	0.074
Left pars opercularis	−8.7	0.020	0.076
Right medial orbitofrontal	−5.5	0.016	0.153
Left supramarginal	−8.6	**0.007**	**0.043**
Right supramarginal	−6.7	0.019	0.171
Cortical thickness			
Left entorhinal	−2.3	0.047	0.055
Left inferior parietal	−2.5	**0.006**	**0.006**
Left superior parietal	−2.3	**0.021**	**0.046**
Right entorhinal	−3.7	**0.017**	**0.005**
Right pars triangularis	−2.4	**0.040**	**0.022**
Subcortical volume			
Left thalamus	−4.5	0.013	0.129
Right thalamus	−4.4	0.014	0.153

We calculated the % change in size of the atlas regions between groups. The column to the right displays *P*-values for these models while additionally including ICV as a covariate. *P*-values in bold are significant also when correcting for ICV.

^a^Interaction between group and sex for these regions.

##### Covariates: age, sex, ICV

**Fig. 1:**
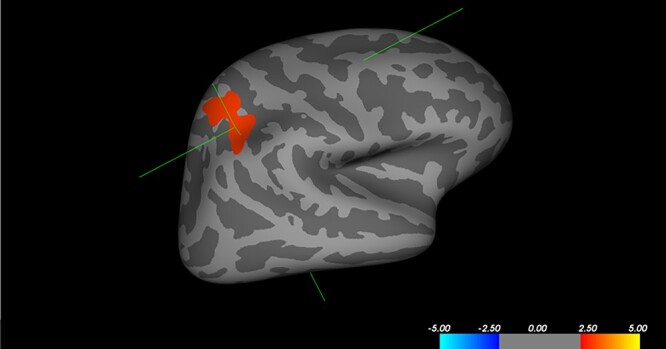
Reduced volume of the right superior parietal cortex in males with AAD. Male participants with AAD (*n* = 19) had reduced volume of the right superior parietal cortex compared to control males (*n* = 31), *t*(max) = 2.72, size = 1166.51 mm^2^, peak Talairach (*X* = 30.6, *Y* = −47.5, *Z* = 44.4), as assessed with vertex-wise analysis in FreeSurfer. The color bar indicates *t*-values, with red indicating positive *t*-values, i.e. increased volume in controls.

When including ICV as a covariate, the main group differences were no longer significant. However, 2 findings remained: (i) the interaction between group and sex for volume of the right superior parietal cortex remained and (ii) males with AAD had smaller volume of this region compared to controls (see Fig. 1). This finding was not replicated using the Desikan–Killiany atlas (but see Table 4 for remaining subthreshold results).

#### White matter microstructure

There were no group differences and no sex-specific effects for any of the white matter microstructure estimates.

### Associations between brain structure, working memory, self-reported executive function problems, and disease-related factors

#### Working memory and self-reported executive function problems

**Fig. 2:**
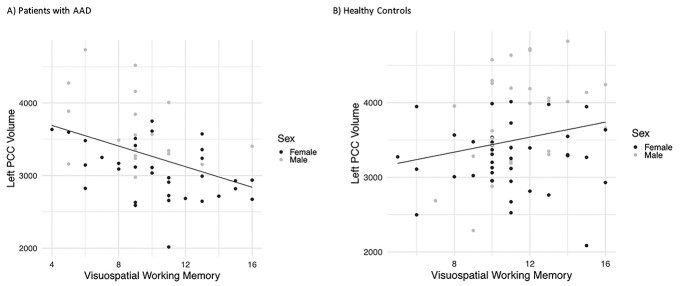
Associations between brain structure and visuospatial working memory. There was a significant interaction between group (patient or control) and volume of the left posterior cingulate cortex (PCC), and performance on a visuospatial working memory test (Span Board forward) (*B* = −0.003, 1 = 0.014). Post hoc tests revealed that in individuals with AAD, those with larger volume of the left PCC performed worse on the task (*B* = −0.003, *P* = 0.016), while in healthy controls, those with larger volumes performed better (*B* = 0.001, *P* = 0.048).

There was a significant interaction between group and volume of the left posterior cingulate cortex for visuospatial working memory forward, even when correcting for ICV (= −0.003, *q* = 0.014). Post hoc tests revealed that, when correcting for ICV, participants with AAD with greater volume of the left posterior cingulate cortex performed “worse” on this task (*B* = −0.003, *P* = 0.016), while healthy controls with greater volume of this region performed better (*B* = 0.001, *P* = 0.048) (see Fig. 2A and B). There were no interactions between group and working memory for white matter microstructure estimates. There were no interactions between group and self-reported executive function problems on BDEFS, and any brain structure estimate.

#### Disease-related factors

##### Covariates: full disease factor model

**Fig. 3:**
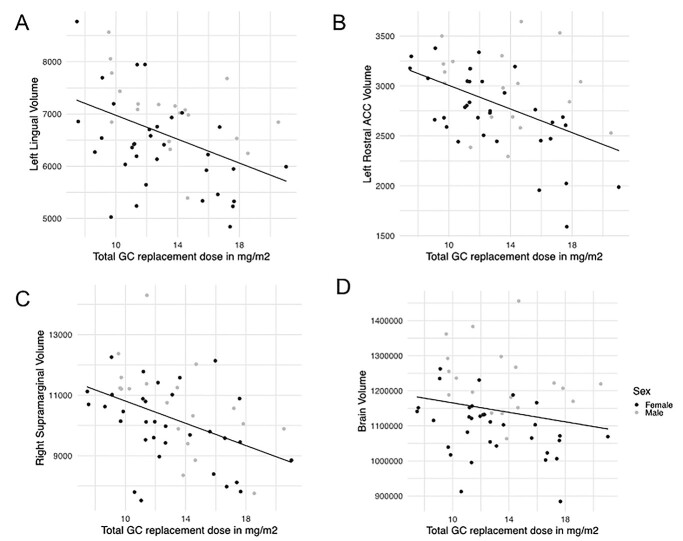
Associations between GC medication dose in mg/m^2^/day and brain volumes. A higher GC replacement dose in mg/m2/day was associated with reduced volume of the (A) left lingual (*B* = −106.28, *q* = 0.045), (B) rostral anterior cingulate (ACC) (*B* = −59.45, *q* = 0.033), and (C) right supramarginal gyrus (*B* = −177.30, *q* = 0.045), but not when correcting for intracranial volume (ICV). D) GC replacement dose in mg/m^2^/day was associated with reduced total brain volume (*B* = −8383, *P* = 0.027), even when correcting for height (*B* = −7124, *P* = 0.45).

There was a positive association between GC replacement dose in mg/m^2^/day and regional brain volumes: individuals with AAD on a higher GC replacement dose in mg/m^2^/day had smaller volume of the left lingual (*B* = −106.28, *q* = 0.045), rostral anterior cingulate (*B* = −59.45, *q* = 0.033), and right supra marginal gyrus (*B* = −177.30, *q* = 0.045) (see Fig. 3). However, these findings did not survive FDR correction when adding ICV as a covariate.

##### Association between GC dose and total brain volume: covariates age, sex

A higher GC replacement dose was associated with smaller total brain volume (without ventricles) (TBV) (*B* = −8383, *P* = 0.027), entailing a reduction of 0.73% TBV (8,383/1,144,326 ^*^ 100), for every mg/m^2^/day increase in GC dose. As medication doses in our patient group ranged between 7.5 and 21 mg/m^2^/day, we may expect at the most around 9.9% (13.5 ^*^ 0.73) difference in total brain volume related to GC medication dosing. GC replacement dose was not associated with number of experienced adrenal crises in the total patient group (*B* = −0.07, *P* = 0.450), but individuals with AAD who had experienced more adrenal crises were more likely to be on MR-HC (*B* = 0.13, *P* = 0.040). There were no sex-specific effects.

### Sensitivity analyses: the relationship between GC replacement doses and brain volumes

#### IR-HC group separately

When assessing the IR-HC group separately, the associations between GC dose and volume of the lingual (*B* = −87.48, *q* = 0.042) and rostral anterior cingulate gyrus (*B* = −69.81, *q* = 0.027) remained when testing the full disease factor model, but not when correcting for ICV. The negative association between GC replacement dose and total brain volume (*B* = −8792.6, *P* = 0.042) also remained within the IR-HC group, correcting for sex and age.

#### Assessment of the impact of patient height on the relationship between GC-dose and TBV

As medication dose is estimated in mg/m^2^/day, size of the person may confound the relationship between GC dose and brain volume, since shorter individuals could have both a relatively higher medication dose and relatively smaller brains. Height in cm was indeed significantly associated with total brain volume (*B* = −7124, *P* = 0.045), but *not* with GC dose/m^2^/day (*B* = −0.08, *P* = 0.382). Moreover, body mass index did not correlate with brain volume or GC dose/m^2^/day. When including height as a covariate, the relationship between total brain volume and medication dose remained significant (*B* = −7124, *P* = 0.045), entailing a 0.62% brain volume reduction, with every increase in mg/m^2^/day, and a maximum expected difference in TBV related to medication use of 8.4% (13.5 ^*^ 0.62).

##### Working memory associated with GC replacement dose

Because we found that a higher GC replacement dose was associated with smaller brain volume, and while smaller PCC volumes were associated with better working memory, we ran this extra analysis. A higher daily GC replacement dose in mg/m^2^/day was associated with better performance on one of the visuospatial working memory tasks (Span Board forward) (*B* = 0.28, *P* = 0.030).

##### White matter microstructure

None of the disease-related factors were associated with the white matter microstructure estimates FA, MD, RD, or AD.

#### Subgroup analyses

There were no differences in any of the brain estimates between individuals with comorbid hypothyroidism and age- and sex-matched healthy controls (*n* = 22 individuals with co-morbid hypothyroidism and *n* = 22 healthy controls).

## Discussion

To the best of our knowledge, this is the first brain imaging study in individuals with AAD. Individuals with AAD had reduced total brain volumes compared to healthy controls, and a higher GC replacement dose in patients correlated with smaller total brain volumes, as well as with smaller volume of the left lingual gyrus, left anterior cingulate cortex, and right supramarginal gyrus. When considering head size, participants with AAD did not have substantial differences in terms of regional cortical thickness, surface area, cortical gray matter volume, and subcortical volumes. However, male patients had smaller volume of the right superior parietal cortex. White matter microstructure was not affected.

According to the methods used in this study, individuals with AAD that were diagnosed at a relatively young age and have been treated with GCs for nearly a decade on average, have reduced brain volume without clear regional specificity. The observed structural changes seem less profound and less region-specific compared to those found in other cortisol-related disorders, such as CAH and Cushing’s disease (Andela et al. 2015; Karlsson et al. 2017; Webb et al. 2018; Van't Westeinde et al. 2019). This may be related to the fact that AAD originates later in life compared to CAH (Gidlöf et al. 2014; Speiser et al. 2018) and has a milder impact on cortisol disturbance relative to Cushing’s disease (Newell-Price et al. 2006; Steffensen et al. 2010). The less substantial structural changes in AAD are in accordance with the finding that our patients perform within the normal range on a variety of cognitive tasks, although females reported to experience problems with executive function in daily life (van’t Westeinde et al. 2022).

Nonetheless, participants with AAD did have 4.3% smaller total brain volumes compared to controls. In addition, although the findings were too small to survive multiple comparisons testing, regions including the inferior and superior parietal cortex, supramarginal gyrus, orbitofrontal cortex, parahippocampal gyri, and the bilateral thalamus were smaller in patients and therefore likely contributed the most to the reduction in total brain volume. The regions driving the effect in AAD, most notably the orbitofrontal cortex and parahippocampal gyri, are known to contain a high density of GC receptors, which might make them more vulnerable (Reul and de Kloet 1985; McEwen and Magarinos 2001; Joëls 2008; Madalena and Lerch 2017). Long-term studies are needed to investigate if these areas become significantly affected with prolonged disease duration. Interestingly, the difference in brain volume is very comparable to that observed in our CAH cohort, namely 4.2% (Van't Westeinde et al. 2019). Moreover, we found the parietal cortex to be affected structurally also in CAH (Van't Westeinde et al. 2019). Our results suggest that the parietal cortex might be particularly sensitive in primary adrenal insufficiency. Indeed, male participants with AAD in the present study did have significantly lower volume of the right superior parietal cortex. In addition, the whole group difference in left supramarginal gyrus volume reduction amounted to 10%, which is along the line of volume change found in Cushing’s disease that can be restored after treatment (Starkman et al. 1999; Andela et al. 2015). The superior parietal cortex is a key node of the visuospatial working memory network (Eriksson et al. 2015), and the high energetic demand of these parietal nodes might make them more vulnerable (Goyal et al. 2014). For example, changes in glucose metabolism in the parietal cortex are correlated with cortisol levels in Cushing’s disease (Liu et al. 2018). Why males would be affected specifically remains to be investigated. Due to the loss of androgens in women, and their self-reported problems with executive function (van’t Westeinde et al. 2022), we had expected to find changes in structure of the brain rather in females as opposed to males. It seems that adrenal androgen loss in women with AAD does not lead to brain structure alterations at this age.

Further, a higher GC replacement dose correlated with reduced volume of the left lingual gyrus, left rostral anterior cingulate cortex and right supramarginal gyrus, as well as with reduced total brain volume. Thus, a higher GC replacement dose could potentially be associated with brain volume reduction, in particular in the 3 areas mentioned. These regions are involved in word processing (Mechelli et al. 2000), directing attention and decision-making (Heilbronner and Hayden 2016), and verbal and visuospatial working memory (Eriksson et al. 2015). These findings are interesting, considering that one of the most consistent cognitive impairments reported in individuals with AAD so far are in verbal learning and memory (Schultebraucks et al. 2015; Henry et al. 2017).

The estimated relationship between GC dose and volume was 0.73% reduced total brain volume for every increase in mg/m^2^/day. With a medication dose ranging between 7.5 and 21 in our cohort, we estimated a maximum difference within the patient group of 9.9% total brain volume. The question remains whether these differences might be clinically relevant, and whether they reflect a maladaptive process, or are part of a compensatory mechanism. High doses of cortisol have been associated with impaired neurogenesis, which may explain the potential mild atrophy in patients on relatively higher doses (Shirazi et al. 2015; Stomby et al. 2016; Morsi et al. 2018). However, in our cohort, a higher medication dose was associated with “better” performance on a visuospatial working memory task. This is in accordance with our previous finding where a higher medication dose was associated with less self-reported problems with executive functioning (van’t Westeinde et al. 2022). In addition, “lower” volume of the left posterior cingulate cortex was associated with “better” performance on this task only in individuals with AAD. It is important to note that bigger is not necessarily always better, and reduced volumes *may* be part of an adaptive mechanism. Alternatively, the reduced overall brain volume, and regional reduction in other areas, might point at divergent effects of GC replacement dose on brain structure and cognitive outcome for this patient group. A higher dose might enable compensation mechanisms to achieve similar performance, while at the same time leading to brain volume loss. Compensatory brain function should be assessed in studies of resting-state and task performance-related functional activity, in particular on the long term. In addition, a reorganization of brain structure might occur that we were not able to detect with the current measures used.

As this is a cross-sectional study, we are unable to draw conclusions about causality. For example, participants with AAD who did not feel well might have been put on a higher medication dose and experienced brain volume reduction prior to increasing the GC dose. There is currently no biomarker that can be used to monitor if the dosing is adequate. Patients may have a variety of reasons to be put on higher or lower doses. For example, some women may request a lower GC dose as they are worried about gaining weight, while others may request a higher dose as it makes them feel better, even though there are no clinical parameters to guide such decisions. Therefore, being on a higher GC dose (in a clinical context) may implicate confounders that could also explain the observed relationship with total brain volume.

Nonetheless, brain volume loss is a potential reason for concern, and long-term follow-up studies are required to test if volume loss leads to substantial differences in brain structure at later ages, whether it affects cognitive abilities and/or mood, or if it leads to accelerated age-related decline.

### Limitations

There are some limitations to consider. First, the groups were not precisely matched on age, which may have diluted the results. GCs have a differential effect on the brain depending on developmental stage (Lupien et al. 2009); thus, the age of diagnosis is likely to influence the impact of having AAD on the brain. Although most of our participants were diagnosed in adulthood, the age of diagnosis varied between 13 and 34 years old. Therefore, the lack of effects of age of diagnosis on brain structure may have been confounded by the fact that the ones diagnosed at a younger age also were younger when tested. Larger studies that allow for sufficiently powered patient groups with different ages of diagnosis are needed. Second, we performed many inferential statistical tests and therefore had to perform strict multiple comparisons corrections that may have led us to miss smaller effects. Indeed, although our results from the vertex-wise analyses were not replicated in the parcellated approach post FDR correction, uncorrected group differences in various brain regions may, nonetheless, be clinically relevant long term. More detailed regional analyses are thus warranted. However, as this was a first-ever brain structure study in this patient cohort, we opted for an exploratory whole-brain approach rather than focusing solely on areas related to already well-established hypotheses about GC effects on the brain. Third, relative to the control group, more images were excluded from the AAD group due to poor image quality. In addition to reducing the power of the study, it suggests that individuals with AAD moved more in the scanner. Since higher levels of motion may be anxiety-driven, it may be that the excluded images belonged to those participants who were most affected. In addition, while we excluded control participants undergoing antidepressant treatment from the study, antidepressant use was allowed in the patient group as mood-related symptoms may be a consequence of AAD. As mood disorders are associated with brain structure, this may have confounded our results (Zhang et al. 2018). Future studies may benefit from including a clinical control group to account for confounders such as comorbid psychiatric disorders.

## Conclusion

Young adults with Addison’s disease have around 4% smaller brain volumes but did not have profound regional changes in structure of the brain, as assessed with the gross voxel-wise whole-brain estimates in this study. These findings are reassuring and indicate that the disease is well managed at this age in Sweden. However, volume loss in association with a higher GC replacement dose might potentially lead to problems later in life, which needs to be studied with long-term follow-up research. In addition, both detailed regional and whole-brain organization analyses are needed to rule out relevant structural and functional alterations in these individuals.

## Supplementary Material

Supplementary_Table_FINAL_snac016

## Data Availability

Restrictions apply to the availability of data generated or analyzed during this study to preserve patient confidentiality or because they were used under license. The corresponding author will on request detail the restrictions and any conditions under which access to some data may be provided.
