# Supplementary material for: Brain structure in autoimmune Addison’s disease
Source: Cereb Cortex. 2022 Oct 13;33(8):4915–26. doi: 10.1093/cercor/bhac389 (PMC10110435; doi:10.1093/cercor/bhac389)
Supplement: Supplementary_Table_FINAL_snac016 [file supplementary_table_final_snac016.docx]

**Supplementary Table 1.** Demographics, medication use and disease characteristics of the individuals with AAD with and without co-morbid hypothyroidism (HT)

|  | **HT, n=22** | **No HT, n=30** | **p-value (HT vs No HT)** |
| --- | --- | --- | --- |
| Sex (female/male) | 16/6 | 17/13 | 0.370 |
| Age (mean (SD), range) | 32.9 (5.9)  19.3-41.8 | 32.2 (6.7)  19.3-41.9 | 0.795 |
| Education (% higher educated) | 54.5 % | 46.7% | 0.779 |
| Parental education (% higher educated) | 36.4% | 46.7% | 0.646 |
| **Type of GC** |  |  |  |
|  |  |  |  |
| IR-HC (n) |  |  |  |
| GC dose (mg/m2/day)^1^ (mean (SD)) | 14.7 (4.1) | 12.5 (2.7) | 0.143 |
| Doses/day (mean (SD)) | 2.6 (0.6) | 2.6 (0.7) | 0.824 |
| MR-HC (n) | 6 | 3 |  |
| GC dose (mg/m2/day)^2^ (mean (SD)) | 13.5 (2.2) | 9.9 (2.4) | 0.095 |
| Doses/day (mean (SD)) | 1.5 (0.8) ^3^ | 1.3 (0.6) | 1 |
| 9-α fludrocortisone, median dose in mg (range) | 0.1 (0.05-0.20) | 0.1 (0.05-0.25) | 0.342 |
| **Disease Characteristics** |  |  |  |
| Age at AAD diagnosis (yrs.), (mean (SD))  median  range | 23.1 (6.3)  22.5  14-34 | 22.7 (6.5)  22  13-34 | 0.860 |
| AAD duration (yrs.), (mean (SD))  median  range | 9.8 (4.5)  10.4  2.5-19 | 9.5 (5.5)  9.3  2.8-24.9 | 0.624 |
| Number of adrenal crises, (mean (SD))  median  range | 4.3 (6.7)  1.5  0-25 | 2.8 (4.0)  1  0-17 | 0.512 |
| **ADHD, Depression & Anxiety** |  |  |  |
| ADHD diagnosis (yes) | 0 | 1 | 1 |
| Antidepressant use (yes) | 2 | 5 | 0.704 |
| MADRS (mean (SD), range) | 9.2 (6.0)  0-25 | 8.2 (6.3)  0-24 | 0.571 |
| HADS Depression (mean (SD), range) | 4.0 (3.4)  0-11 | 3.5 (3.2)  0-10 | 0.464 |
| HADS Anxiety (mean (SD), range) | 6.9 (3.0)  3-16 | 7.6 (5.0)  2-20 | 0.541 |

**^1^** Total IR-HC dose per day in mg per m2 of body surface (total mg/sqrt(cm*kg/3600))

**^2^** Total IR-HC equivalence dose per day in mg per m2 of body surface (total mg/sqrt(cm*kg/3600))

**^3^** Some participants on MR-HC take additional IR-HC doses on demand

HT = individuals with AAD with co-morbid hypothyroidism, No HT= individuals with AAD without co-morbid hypothyroidism.
P-values indicate comparisons between individuals with AAD with and without co-morbid hypothyroidism, Wilcoxon tests for non-parametric data were used for the comparison of age at testing, GC dose, number of doses, fludrocortisone intake, age of AAD diagnosis, AAD duration and number of adrenal crises. Chi-square tests were used for the relative number of participants with an ADHD diagnosis and using antidepressants, and subject and parent level of education, linear models are used for the comparison on MADRS, HADS Depression and HADS Anxiety as in these models sex and age were included as covariates.
